# Plant Resource Availability of Medicinal *Fritillaria* Species in Traditional Producing Regions in Qinghai-Tibet Plateau

**DOI:** 10.3389/fphar.2017.00502

**Published:** 2017-08-07

**Authors:** Dongdong Wang, Xiong Chen, Atanas G. Atanasov, Xiao Yi, Shu Wang

**Affiliations:** ^1^Department of Molecular Biology, Institute of Genetics and Animal Breeding of the Polish Academy of Sciences Jastrzębiec, Poland; ^2^Department of Pharmacognosy, West China College of Pharmacy, Sichuan University Chengdu, China; ^3^Department of Pharmacognosy, Faculty of Life Sciences, University of Vienna Vienna, Austria; ^4^The Public Health Clinical Center of Chengdu Chengdu, China; ^5^Department of Vascular Biology and Thrombosis Research, Center for Physiology and Pharmacology, Medical University of Vienna Vienna, Austria; ^6^Luzhou Products Quality Supervision & Inspection Institute Luzhou, China

**Keywords:** Bulbus Fritillariae Cirrhosae, plant resource, total alkaloids, traditional medicine, plant morphology, artificial cultivation, Qinghai-Tibet Plateau

## Abstract

The genus *Fritillaria* (Liliaceae) comprises of ~140 species of bulbous perennials, which are distributed in the temperate zone of the Northern Hemisphere. *Fritillaria* species have attracted much attention because of their commercial value, partly as ornamental plants but principally as a source of material for use in traditional medicine. The use of *Fritillaria* extracts is well established in some countries in Eastern Europe (e.g., Turkey), and Asia (e.g., China, Japan). In traditional Chinese medicine, the medicinal *Fritillaria* species is called Bulbus Fritillariae Cirrhosae (BFC), which has been used as a traditional medicine for thousands of years. However, to the best of our knowledge, there are no reports on resource investigation of plants of BFC in the last ten years. In this study, we chose 32 traditional producing regions in Qinghai-Tibet Plateau to perform an investigation on resource availability of BFC. In five sites we did not find any plants of BFC. Results show that the average number of the plants of BFC per quadrat in 26 sites was less than 7, and the average resource density was <22 mg/m^2^. Habitat types and plant morphology of BFC plants were recorded. Our investigation shows that the area for artificial cultivation of BFC is larger than 400 hm^2^ and productivity was higher than 180 t. In addition, the total alkaloid contents of samples from cultivated bases and plantations are higher than that from wild fields. This study suggests that the wild populations of BFC are still at the risk of depletion. Artificial cultivation of BFC might be an important way to resolve the current contradiction between resource protection and resource utilization. In addition, identifying the closest European relatives of the *Fritillaria* species used in traditional medicine may resolve this contradiction.

## Introduction

The genus *Fritillaria* (Liliaceae) comprises of ~140 species of bulbous perennials (Day et al., 2014), which are distributed in the temperate zone of the Northern Hemisphere and currently divided into eight subgenera (Rix et al., 2001). *Fritillaria* species have attracted much attention because of their commercial value, partly as ornamental plants but principally as a source of material for use in traditional medicine (Day et al., 2014). The use of *Fritillaria* extracts is well established in some countries in Eastern Europe (e.g., Turkey; Atta ur et al., 1994; Day et al., 2014).

Locals of Çatak in Turkey use some *Fritillaria* species in treatment of several diseases, for example wound healing (Mukemre et al., 2015). The medicinal use of *Fritillaria* species is also well established in China, the Himalayas (India, Nepal, and Pakistan), Japan, Korea, and Southeast Asia (Day et al., 2014). In traditional Chinese medicine, the medicinal *Fritillaria* species is called Bulbus Fritillariae Cirrhosae (BFC), also known by the Chinese name “Chuan Bei Mu,” which has been used as a traditional medicine for thousands of years.

BFC was recorded in the traditional Chinese medicine book *Shen Nong Ben Cao Jing* (*The Divine Farmer's Materia Medica Classic*), which is one of the earliest classic medical book written in the Eastern Han Dynasty (25–220; Wang, 2012). In the *Chinese Pharmacopoeia* (2010 Edition), the botanical origins of BFC include six species of the *Fritillaria* genus: *Fritillaria cirrhosa* D. Don, *F. unibracteata* Hsiao et K.C. Hsia, *F. przewalskii* Maxim, *F. delavayi* Franch, *F. taipaiensis* P. Y. Li and *F. unibracteata* Hsiao et K. C. Hsia var. *wabuensis* (S. Y. Tang et S. C. Yue) Z. D. Liu, S. Wang et S. C. Chen (Pharmacopoeia, 2010).

BFC is used as a folk medicine because of its remarkable antitussive, expectorant and antiasthmatic activities (Pharmacopoeia, 2010). Previous studies indicated that chemical constituents of BFC mainly include steroidal alkaloids, saponins, terpenoids, and glycosides (Hao et al., 2013). It is believed that alkaloids are the major biological active constituents (Li et al., 2013; Wang et al., 2014a). Recent pharmacological studies found that alkaloids from BFC exhibit remarkable antitussive, expectorant, anti-inflammatory effects (Wang et al., 2011, 2012), antiasthmatic effect (Yan, 2005), hypotensive property (Kang et al., 2004), anti-bacterial activity (Li et al., 2005), and antitumor effect (Wang D. D. et al., 2014; Wang et al., 2014b, 2015).

Currently, there are 400 manufacturers producing over 200 kinds of preparations containing BFC, because of its obvious effects (Li et al., 2012). The production of medicinal preparations containing *F. cirrhosa* is an industry with an estimated value of US $400 million per year (Day et al., 2014). At present, BFC is mainly harvested from the wild fields. Production of BFC has fallen short of consumption (Wang et al., 2014a). The price of BFC was beyond $260 per kilogram in 2014, almost nine-folds of that in 2004. The growing price of BFC resulted in over-exploitation. Nowadays, these used species in *Fritillaria* genus have been classified as precious, rare and threaten species by Endangered Species Scientific Commission of China in 2012. Some other countries (e.g., Burma, Turkey) are involved in supplying the increasing demand (Day et al., 2014).

However, to the best of our knowledge, there are still no studies on wild resource of plants of the traditional medicine BFC. The present study focused on investigation of the current resource status of BFC in traditional BFC-producing regions in Sichuan, Chongqing, and Yunnan provinces in Qinghai-Tibet Plateau.

## Materials and methods

### Sampling sites

The literature survey was performed to obtain information about distribution of the plants of BFC, which would be useful for choosing the sampling sites. According to *Flora of China, Flora of Sichuan Province*, and *Flora of Yunnan Province* and previous studies (Luo and Chen, 1996; Liu et al., 2008), 32 sites in Sichuan, Chongqing, and Yunnan provinces, which are the traditional producing regions of BFC, were chosen to perform a survey on plant resource of BFC during 2013–2014 (Table 1). In every site, there was one experienced and local herbalist who helped us to carry out resource investigation.

**Table 1:** GPS data and species in different sites, and the content of total alkaloids of the samples from different sites.

	**County**	**The field site**	**Latitude**	**Longitude**	**Altitude (m)**	**Species**	**Content of total alkaloids (%)**
Sichuan	Kangding	Xingduqiao Town, Waze Village, Enwei Plateau Medicinal Plant Fostering Base	N30.0637	E101.5827	3530	*F. cirrhosa*	0.300 ± 0.026
	Daofu	Weita Village	N31.3417	E101.1594	3599	*F. cirrhosa*	0.043 ± 0.002
		Yinen Village	N31.4261	E101.1043	3781	*F. cirrhosa*	0.023 ± 0.002
		Jiazong Village	N31.0910	E101.2119	4243	*F. cirrhosa*	0.023 ± 0.002
	Litang	Qudeng Village	N30.1313	E100.0629	4134	*F. cirrhosa*	0.025 ± 0.004
	Yajiang	Milong Village, Beihou Cun	N29.9444	E101.0323	2569	*F. cirrhosa*	0.124 ± 0.010
		Milong Village, Mashizi Cun	N29.9752	E101.1848	3909	*F. unibracteata*	0.175 ± 0.017
		Milong Village, Lasang Mountain	N29.9515	E101.2083	4503	*F. delavayi*	0.006 ± 0.001
	Songpan	Shanba Village, Duoji mountain	N33.0342	E103.7133	3502	*F. unibracteata*	0.054 ± 0.014
		Sedi Village, Geliping	N32.9613	E103.3471	3841	*F. unibracteata*	0.072 ± 0.008
		Shuijing Village, New Lotus Chuanbeimu Cultivation Base	N32.9574	E103.7113	3321	*F. wabuensis*	0.209 ± 0.006
	Hongyuan	Hongxi Village, Fumin Chuanbeimu Cultivation Base	N32.8806	E102.6102	3510	*F. unibracteata*	0.072 ± 0.008
		Hongxi Village, Luosang Chuanbeimu Cultivation Base	N32.7782	E102.5625	3530	*F. unibracteata*	0.034 ± 0.003
		Hongxi Village, Caimugou	N32.7420	E102.5745	3578	*F. unibracteata*	0.034 ± 0.002
	Aba	Jiaerduo Village, Nuoerdang Cun	N33.2103	E101.4662	3723	*F. unibracteata*	0.036 ± 0.005
		Jiaerduo Village, Fenshui Ling	N33.4153	E101.4985	3693	*F. unibracteata*	0.030 ± 0.006
	Rangtang	Gangmuda Village, Jiudaoguai	N32.3070	E101.0647	3898	*F. unibracteata*	0.027 ± 0.002
		Gangmuda Village, Gage Mountain	N32.3067	E101.0649	3915	*F. unibracteata*	0.025 ± 0.004
	Luhuo	Renda Village, Guobalong	N31.2178	E100.8387	3119	-	-
		Renda Village, Ribudike	N31.2161	E100.7911	3826	-	-
	Mao	Songpinggou Village, New Lotus Chuanbeimu Cultivation Base	N32.1988	E103.4953	3729	*F. unibracteata*; *F. wabuensis*	0.249 ± 0.003
	Xiangcheng	Shuiwa Village	N29.1451	E100.0675	4644	-	-
		Ranwu Village	N28.3141	E99.7467	3887	-	-
Yunnan	Shangri-la	Kari Mountain	N27.8149	E99.7053	3352	-	-
	Lijiang	Hutiaoxia Village	N26.9904	E100.0665	3857	*F. cirrhosa*	0.140 ± 0.012
		Dadong Village, Haizi Cun	N27.0595	E100.2768	3088	*F. cirrhosa*	0.058 ± 0.017
		Dadong Village, Lianggushui Mountain	N27.0260	E100.2930	3131	*F. cirrhosa*	0.068 ± 0.014
Chongqing	Wuxi	Lanying Village, Xian Cun, Zhonghoujiao	N31.4043	E109.8737	1658	*F. taipaiensis*	0.148 ± 0.015
		Lanying Village, Xian Cun, Balicao	N31.4066	E109.8831	1704	*F. taipaiensis*	0.101 ± 0.013
		Lanying Village, Xian Cun, Huangcaoping Chuanbeimu Cultivation Base	N31.4165	E109.9238	2074	*F. taipaiensis*	0.303 ± 0.005
	Chengkou	Mingzhong Village	N31.7381	E108.8206	2786	*F. taipaiensis*	0.179 ± 0.016
Qinghai	Jiuzhi	Ningyou Village	N33.4154	E101.4990	3696	*F. unibracteata*	0.049 ± 0.017

*The measurement of content of total alkaloids was conducted in triplicate. Data were reported as mean ± standard error of mean (SEM) (n = 3)*.

### Field investigation

The field investigation was carried out in May, June, or July in 2013 and 2014. Field investigation was designed to assess the distribution, density and abundance of the plants of BFC (Delgado-Lemus et al., 2014). In order to estimate their distribution, the coordinate positions were firstly recorded using a Magellan Triton GPS400 in each site (Delgado-Lemus et al., 2014). The quadrat investigation was performed to assess the density and abundance of the plants of BFC as described previously (Zhang et al., 2015). Series of squares (quadrats) with a set size (4 × 4 m) were placed in each site. The mean density of the plants of BFC per quadrat was the number of the plants of BFC per quadrat. The resource density of the quadrat was calculated as: Resource density = total weight of bulbs/quadrat area (g/m^2^).

### Collection of plant specimens and bulbs of BFC

At each site, 5–15 plants were collected as specimens during field investigation. The specimens were prepared in the medicinal plants Herbarium of West China College of Pharmacy. The specimens were examined and identified by experts, and the voucher specimens with labels were deposited at the medicinal plants Herbarium. At each site, photographic records were taken to capture the field sites, growth habitat, and morphology of plants (Chekole et al., 2015). We also collected the bulbs from these sites. All samples were subjected to the described determination of content of the total alkaloid.

### Artificial cultivation survey

In this study, we visited six artificial cultivated bases and two individual cultivated plantations. The specific information about area, production, and market value were gathered by inquiring, personal interviewing, or direct surveying (Zhang et al., 2015).

### Measurement of content of total alkaloid

The total alkaloid content was determined according to the previously described acid dye colorimetric method (Wang D. D. et al., 2014; Li et al., 2015). For alkaloid standard, a stock standard solution (0.1 mg/ml) of imperialine (in chloroform) was prepared and the stock solution was diluted to different working concentrations (0.0, 1.0, 2.0, 4.0, 8.0, 10.0 μg/ml).

A certain amount (about 2.0 g) of powdered sample was weighed, immersed in 3 ml of ammonia for 1 h, and then extracted under reflux with 40 ml of chloroform-methanol (3:1) solution for 2 h. The solution was filtered, and diluted to a final volume of 50 ml with chloroform-methanol solution. A certain volume (3–5 ml) of the solution was pipetted and evaporated, and the residue was dissolved in chloroform (10 ml) to get the sample solution. According to the absorption spectra of the standard solutions, 412 nm was chosen as the detection wavelength.

To measure total alkaloid content, 10 ml of each sample or working concentrations of standard solution was transferred into a separate funnel. Five milliliters of distilled water and 2 ml of bromocresol green buffer solution were added into it and shaken for 2 min. After 30 min, the chloroform layer was separated and collected. The absorbance of chloroform fraction was measured at 412 nm using Alpha-1900PC UV–Vis spectrophotometer (Shanghai Lab-Spectrum Instruments Co., Ltd., China). The experimental results were expressed as mean ± standard error of mean (SEM).

## Results

### Resource distribution and density

**Figure 1:**
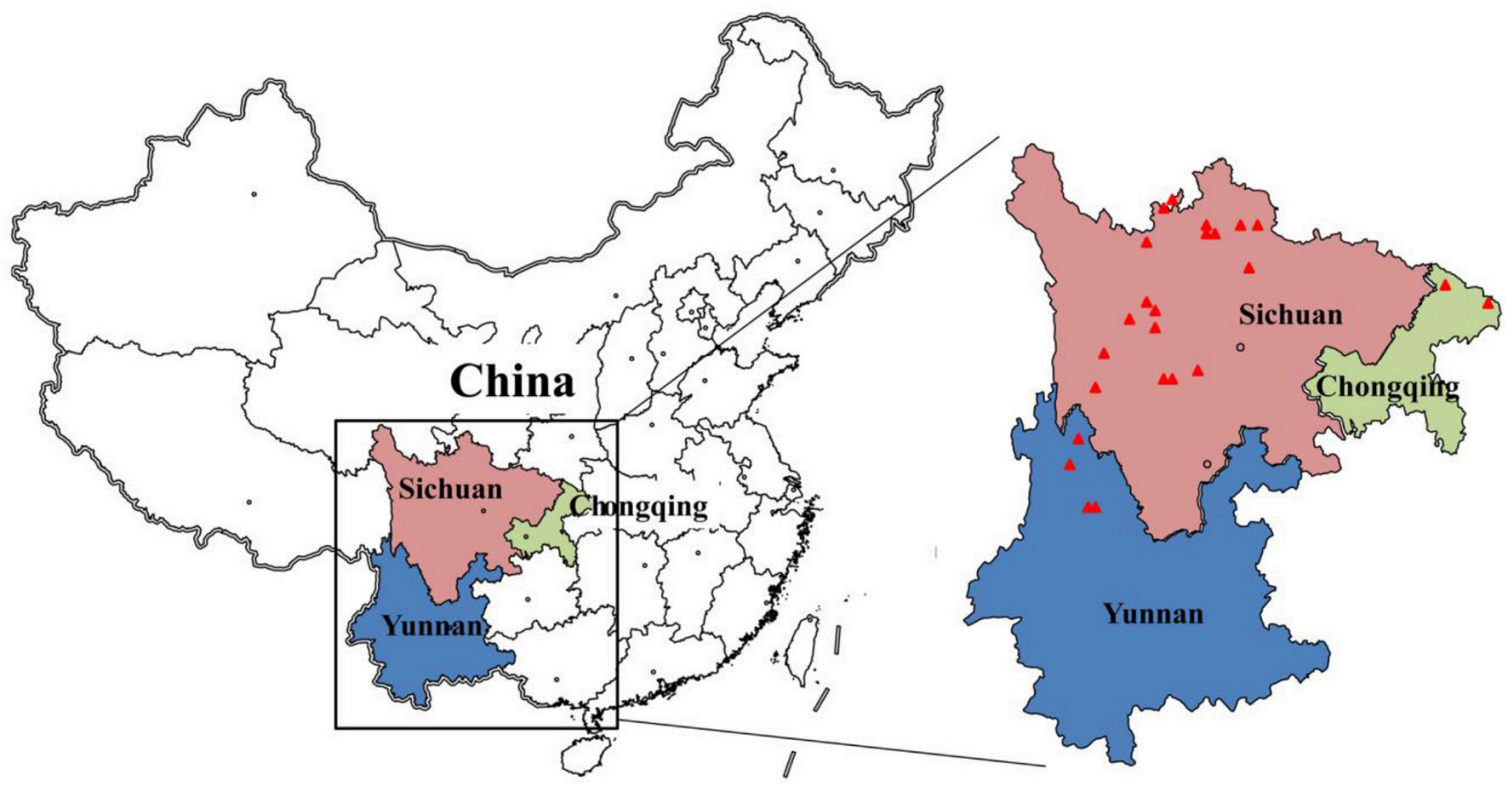
Location of study areas. The red triangle shows the investigation sites. The traditional producing-regions of Bulbus Fritillariae Cirrhosae (BFC) are mainly distributed in Sichuan, Chongqing, and Yunnan Provinces in Southwest China.

The distribution of wild plants of BFC is shown as Figure 1 and Table 1. To investigate the resource of BFC, we chose 32 traditional producing regions of BFC. In five sites investigated no BFC plants could be found. These sites are Guobalong and Gage Mountain in Renda Village in Luhuo county, Shuiwa Village and Ranwu Village in Xiangcheng county in Sichuan Province, Kari Mountain in Shangri-la in Yunnan province. The sites where plants of *F. cirrhosa* were found are Kangding, Daofu, Litang, Yajiang counties in Sichuan province and Lijiang county in Yunnan province (Table 1). The sites where plants of *F. unibracteata* were found are Yajiang, Songpan, Hongyuan, Aba, Rangtang, Litang, Mao counties in Sichuan province and Jiuzhi county in Qinghai province (Table 1). The sites where plants of *F. wabuensis* were found are Songpan and Mao counties in Sichuan province. The sites where plants of *F. taipaiensis* were found are Wuxi and Chengkou counties in Chongqing province (Table 1). We only found plants of *F. delavayi* in Yajiang county in Sichuan province. From the Table 1, the main species of BFC occurring in Sichuan province are *F. cirrhosa, F. unibracteata*, and *F. wabuensis*. The main species in Yunnan province is *F. cirrhosa*. The main species in Chongqing province is *F. taipaiensis*.

Table 2 shows the mean density of the plants of BFC per quadrat and resource density. The average number of the plants of BFC per quadrat is <7 except for the site in Ningyou Village in Jiuzhi county, Qinghai province. In addition, the average resource density is <22 mg/m^2^ except for the sites in Lasang Mountain in Milong Village in Yajiang county, Sichuan province and Ningyou Village in Jiuzhi county, Qinghai province. The data reconfirm that the species in the *Fritillaria* genus are still at risk of depletion. The reason why the average resource density in Lasang Mountain in Yajiang county is higher than other sites may be that the species in this site is *F. delavayi* which price is much less than the other species. The reason for that in Ningyou Village in Jiuzhi county may be that this site is one of the main producing regions of Chinese Caterpillar Fungus, with a much higher price than FBC.

**Table 2:** Density of original plants of Bulbus Fritillariae Cirrhosae and resource density of Bulbus Fritillariae Cirrhosae in the quadrats.

**Province**	**County**	**The field site**	**Density of plants (the number/quadrat)**	**Resource density (mg/m^2^)**
Sichuan	Daofu	Weita Village	2.23 ± 0.76	8.44 ± 0.76
		Yinen Village	1.06 ± 0.82	4.13 ± 0.82
		Jiazong Village	1.77 ± 0.98	7.11 ± 0.98
	Litang	Qudeng Village	4.73 ± 0.58	17.22 ± 0.58
	Yajiang	Milong Village, Beihou Cun	0.75 ± 0.67	2.95 ± 0.67
		Milong Village, Mashizi Cun	1.25 ± 0.40	5.39 ± 0.40
		Milong Village, Lasang Mountain	4.76 ± 0.76	38.30 ± 0.76
	Songpan	Shanba Village, Duoji mountain	3.23 ± 0.29	9.95 ± 0.29
		Sedi Village, Geliping	3.33 ± 0.77	10.56 ± 0.77
	Hongyuan	Hongxi Village, Caimugou	4.66 ± 0.32	16.48 ± 0.32
	Aba	Jiaerduo Village, Nuoerdang Cun	5.75 ± 0.52	17.43 ± 0.52
		Jiaerduo Village, Fenshui Ling	6.56 ± 0.53	21.73 ± 0.53
	Rangtang	Gangmuda Village, Jiudaoguai	2.67 ± 0.82	5.69 ± 0.82
		Gangmuda Village, Gage Mountain	1.75 ± 0.86	5.52 ± 0.86
Yunnan	Lijiang	Hutiaoxia Village	3.67 ± 0.46	16.52 ± 0.46
		Dadong Village, Haizi County	2.34 ± 0.91	8.69 ± 0.91
		Dadong Village, Lianggushui Mountain	2.55 ± 0.95	11.17 ± 0.95
Qinghai	Jiuzhi	Ningyou Village	21.88 ± 0.49	64.40 ± 0.49

*Data of density of original plants and resource density were reported as mean ± standard error of mean (SEM)*.

### Habitat types

**Figure 2:**
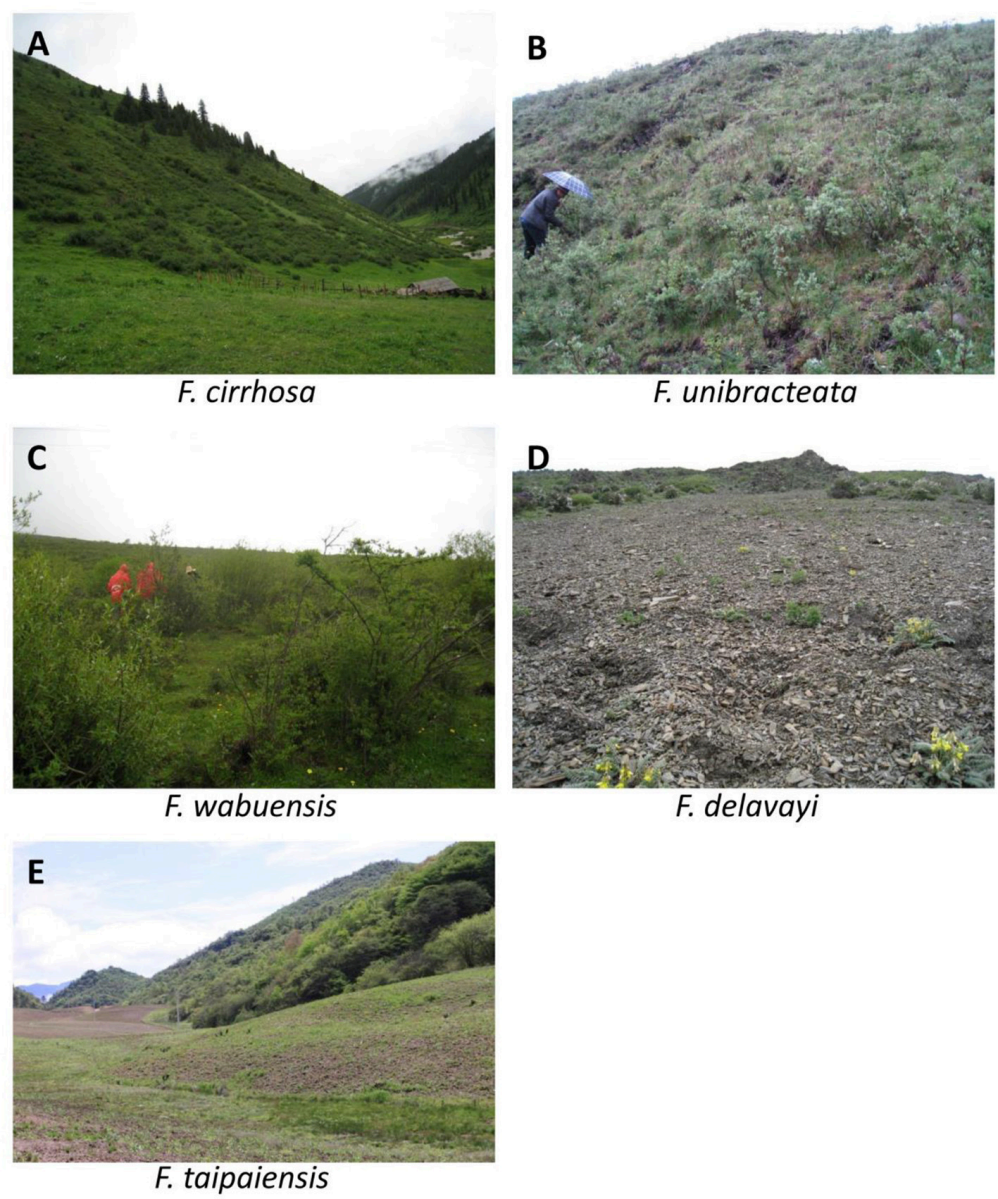
Habitat types of plants of Bulbus Fritillariae Cirrhosae (BFC). **(A)** Habitat type of plants of *F. cirrhosa*; **(B)** Habitat type of plants of *F. unibracteata*; **(C)** Habitat type of plants of *F. wabuensis*; **(D)** Habitat type of plants of *F. delavayi*; **(E)** Habitat type of plants of *F. taipaiensis*.

Plants of *F. cirrhosa* grow under forests, in alpine thickets, or on meadows, and flood lands. They are usually found on shady and moist slopes of valley (Figure 2A). The altitude range of occurrence is from 2,500 to 4,600 m. All altitude data were collected by using Magellan Triton GPS400. The plants of *F. unibracteata* are usually discovered in moist places of thickets or meadows (Figure 2B). Their altitude range is from 3,200 to 4,700 m. Plants of *F. wabuensis* grow under forests, or in alpine thickets (Figure 2C) and the altitude range of their occurrence is from 2,500 to 3,500 m. The plants of *F. delavayi* are usually discovered in sandy and gravelly places or on flood lands (Figure 2D). Their altitude range is often above 4,000 m. Plants of *F. taipaiensis* grow under forests, in hill thickets, or on grassy slopes (Figure 2E). Their altitude range is from 1,500 to 3,200 m. The habitat types of plants of *F. cirrhosa* and *F. unibracteata* are almost the same. The altitudes of plants of *F. taipaiensis* and *F. wabuensis* are lower than that of *F. cirrhosa* and *F. unibracteata*, and altitude of plants of *F. taipaiensis* is the lowest. *F. taipaiensis* and *F. wabuensis* are thus most suitable for artificial cultivation. The plants of *F. delavayi* grow in relatively harsh conditions with higher altitude, lower temperature and lower humidity.

### Plant morphology

**Figure 3:**
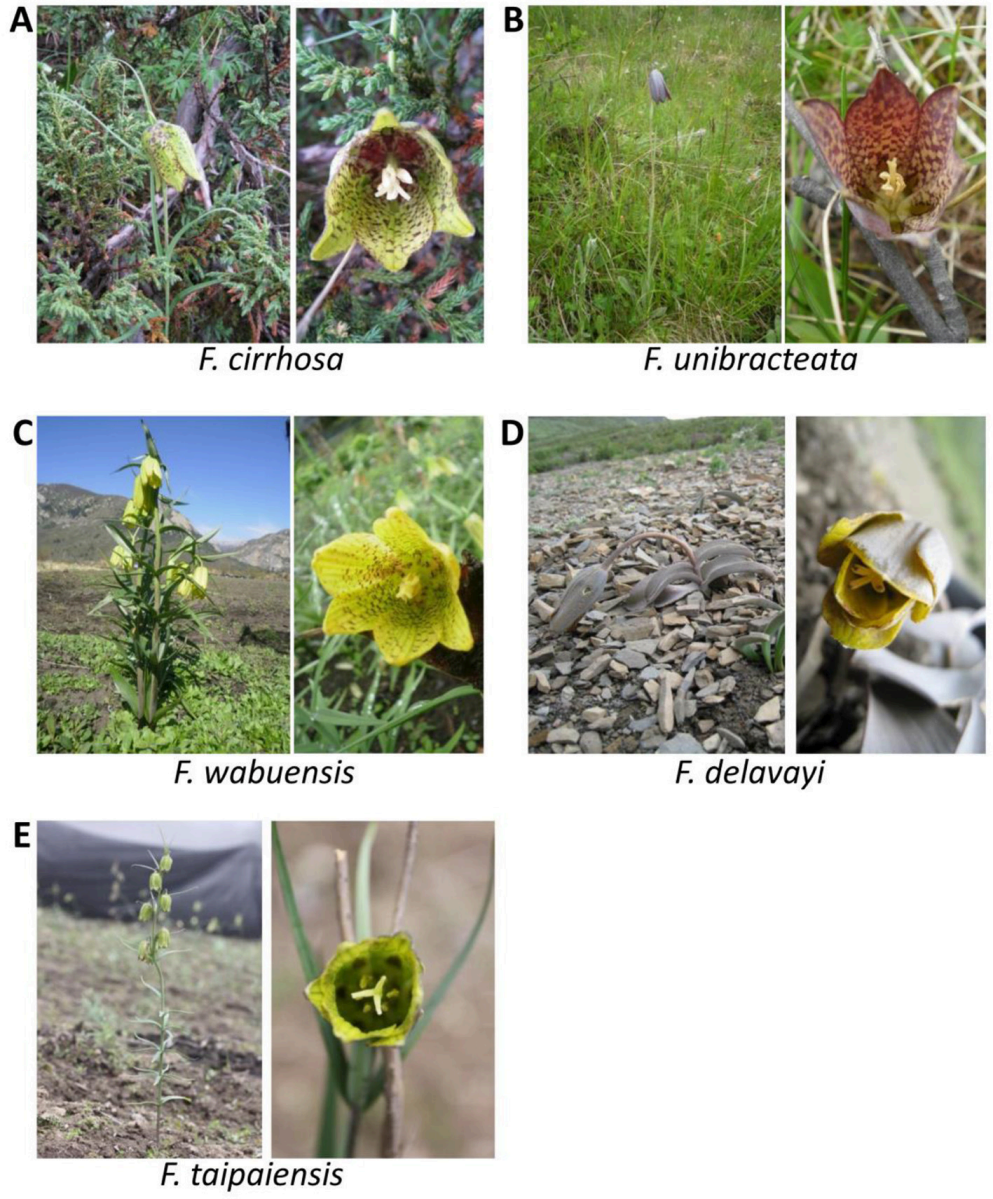
Plants of Bulbus Fritillariae Cirrhosae (BFC). **(A)** Plants of *F. cirrhosa*; **(B)** Plants of *F. unibracteata*; **(C)** Plants of *F. wabuensis*; **(D)** Plants of *F. delavayi*; **(E)** Plants of *F. taipaiensis*.

*F. cirrhosa* (Cheng and Helen, 2000): As shown in Figure 3A, height range of stem is 15–65 cm. Seven to eleven leaves are opposite or sometimes 3- or 4-whorled and alternate. Shape of leaf blade is linear to linear-lanceolate (4–12 cm × 3–15 mm), which apex is often curved or cirrose. The inflorescence is (1-3)-flowered with 3 bracts, whose apex is curved or cirrose. Flowers are bell-shaped, usually nodding. The pedicel is much shorter than tepals. Tepals are yellow or yellowish green, slightly or heavily spotted or tessellated with purple, usually oblong-elliptic (3–5 × 1.2–1.8 cm). Nectaries are elliptic to ovate (3–5 × 2–3 mm), projecting abaxially. Length of stamens is 2–3 cm, filaments sometimes are slightly papillose. The style is 3-lobed with 3–5 mm lobes. Capsule is narrowly winged, and wings are 1–1.5 mm wide.

*F. unibracteata* (Cheng and Helen, 2000): As shown in Figure 3B, height range of stem is 15–50 cm. There are five to seven leaves, and the basal 2 leaves are usually opposite, others are opposite or alternate. The shape of leaf blade is linear to linear-lanceolate (3.6–5.5 cm × 3–5 mm), apex is not curved or cirrose. Inflorescence is (1-3)-flowered with 1 bract whose apex is acuminate. Flowers are bell-shaped, usually nodding. Pedicel is rather long. Tepals are blackish purple, tessellated with yellowish brown or sometimes with a colored, V-shaped stripe near apex. Nectaries are inconspicuous or strongly projecting abaxially. Length of stamens is 1.2–1.5 cm, filaments sometimes are papillose. The style is scarcely or shortly lobed with 0.5–2 mm lobes. The capsule is narrowly winged, and wings are about 1.1 mm wide.

*F. wabuensis* (Cheng and Helen, 2000): As shown in Figure 3C, height range of stem is 50–115 cm. There are 12–28 leaves, and the basal 2 leaves are usually opposite, others are whorled or alternate. Shape of leaf blade is linear-lanceolate (6.5–16.5 cm × 6–25 mm), the apex is not curved or cirrose. The inflorescence is (1-7)-flowered with 1-4 bracts whose apex is acuminate. Flowers are bell-shaped, usually nodding. Tepals are yellowish green, spotted with or without blackish purple. Nectaries are elliptic to ovate (5–8 × 2–3 mm), projecting abaxially. The style is 3-lobed with 3–5 mm lobes. The capsule is narrowly winged, and wings are 1–2 mm wide.

*F. delavayi* (Cheng and Helen, 2000): As shown in Figure 3D, height range of stem is 10–35 cm. Five to seven leaves are closely arranged in middle or distal part of the stem, alternate or subopposite. The shape of the leaf blade is ovate or ovate-elliptic (2–7 × 1–3 cm), apex often is obtuse or rounded. The inflorescence is 1-flowered without bract. Flowers are bell-shaped, usually nodding. The pedicel is long. Tepals are yellowish, spotted or tessellated with reddish brown, narrowly elliptic or oblong-elliptic (3.2–4.5 × 1.2–1.8 cm). Nectaries are inconspicuous. Length of stamens is 1.6–2.2 cm, filaments are glabrous. Anthers are basifixed. The style is 3-lobed with 0.5–5 mm lobes. The capsule is narrowly winged.

*F. taipaiensis* (Cheng and Helen, 2000): As shown in Figure 3E, height range of stem is 20–100 cm. Five to twenty leaves are usually opposite or sometimes middle and distal ones also whorled and alternate. The shape of the leaf blade is linear to linear-lanceolate (5–13 cm × 3–12 mm), apex often is not curved. The inflorescence is (1-3)-flowered with 3 bracts whose apex is often curved. Flowers are bell-shaped, usually nodding. The pedicel is 2–4 cm. Tepals are yellowish green, densely spotted purple, narrowly oblong or obovate-oblong (2.5–5 × 0.6–1.8 cm). Nectaries are slightly projecting abaxially. Length of stamens is about 3/5 as long as repals, filaments are slightly papillose distally. The style is 3-lobed with 2–4 mm lobes. The capsule is winged, and wings are 0.5–2.0 mm wide.

### Artificial cultivation

**Figure 4:**
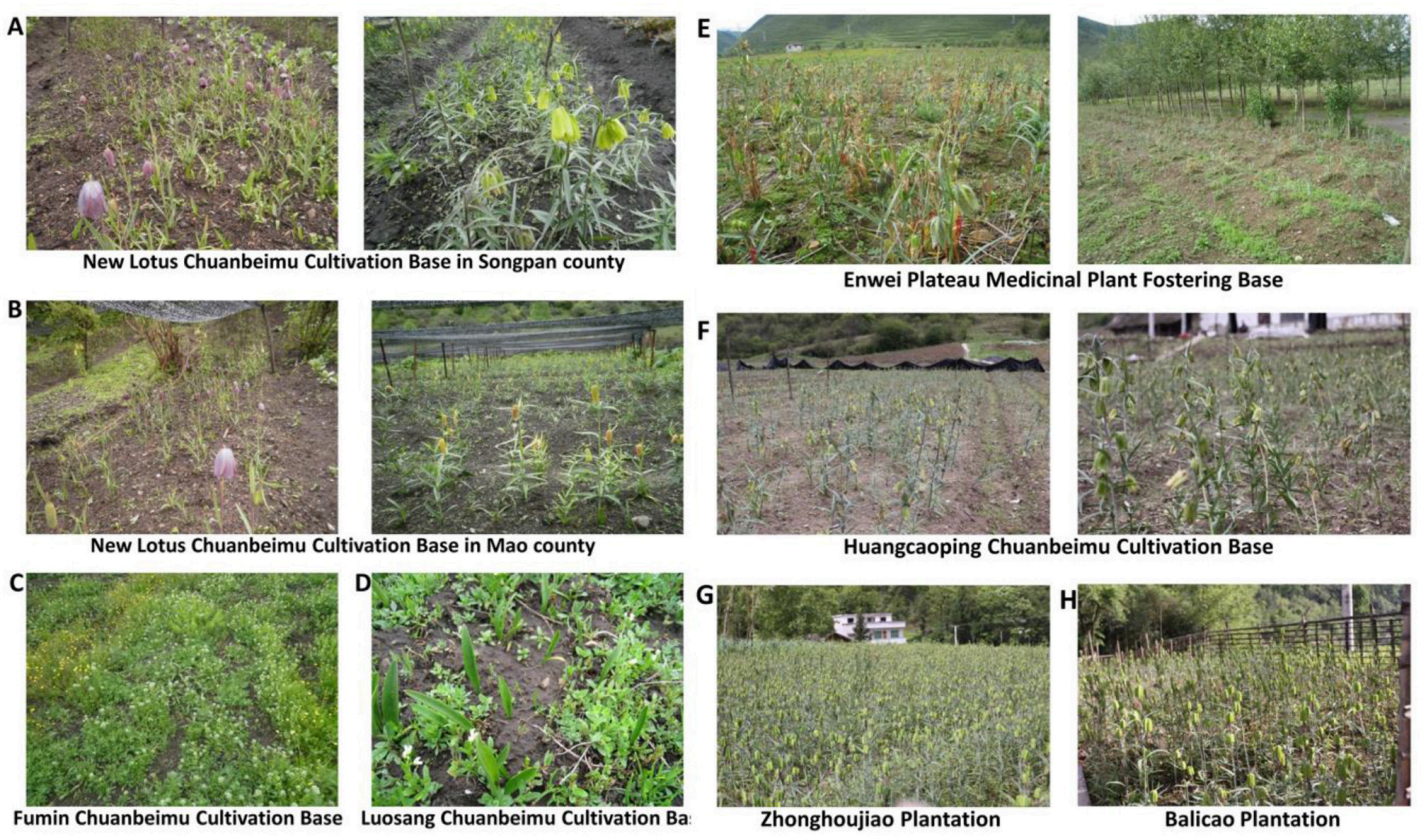
The artificial cultivated bases and individual cultivated plantations. **(A)** New Lotus Chuanbeimu Cultivation Base in Songpan county; **(B)** New Lotus Chuanbeimu Cultivation Base in Mao county; **(C)** Fumin Chuanbeimu Cultivation Base; **(D)** Luosang Chuanbeimu Cultivation Base; **(E)** Enwei Plateau Medicinal Plant Fostering Base; **(F)** Huangcaoping Chuanbeimu Cultivation Base; **(G)** Zhonghoujiao Plantation; **(H)** Balicao Plantation.

Because production of BFC obtained from the wild field has fallen short of consumption and the price is growing higher in recent decades, the artificial cultivation of BFC has been gradually developed by the traditional Chinese medicinal cultivation companies (Wang et al., 2014a). In this work, we investigated six artificial cultivated bases and two individual cultivated plantations (Figure 4). As shown in Table 3, the cultivated bases or plantations are located in Kangding, Mao, Songpan, and Hongyuan counties in Sichuan province, and Wuxi county in Chongqing province. The species in cultivation include *F. cirrhosa, F. unibracteata, F. wabuensis*, and *F. taipaiensis*, which grow in relatively easier habitats compared to other species. We also found that lots of farmers in Wuxi county cultivate *F. taipaiensis* in gardens or surrounding the home yard. The income of selling bulbs of *F. taipaiensis* is one of the important economic sources for the region. Enwei Plateau Medicinal Plant Fostering Base and New Lotus Chuanbeimu Cultivation Base are the two largest bases among the investigated bases covering an area of about 400 hm^2^ and their productivity is up to 180 t. However, the production of BFC from artificial cultivation is far short of what is needed (Liu et al., 2008). So, at present wild populations is still the main source of BFC in the market, which may further aggravate BFC wild resource depletion.

**Table 3:** The details of the artificial cultivated bases and home cultivated plantations.

**Name of bases or plantation**	**Species**	**Area (m^2^)**	**Production (t)**	**Income (dollar)**
Enwei Plateau Medicinal Plant Fostering Base	*F. cirrhosa*	180 × 10^4^	20	12 × 10^6^
New Lotus Chuanbeimu Cultivation Base in Shuijing Village	*F. wabuensis* and *F. unibracteata*	10 × 10^4^	8	4.0 × 10^6^
Fumin Chuanbeimu Cultivation Base	*F. unibracteata*	20 × 10^4^	14	8.1 × 10^6^
Luosang Chuanbeimu Cultivation Base	*F. unibracteata*	0.12 × 10^4^	0.09	0.04 × 10^6^
New Lotus Chuanbeimu Cultivation Base in Songpinggou Village	*F. wabuensis* and *F. unibracteata*	200 × 10^4^	160	80.6 × 10^6^
Huangcaoping Chuanbeimu Cultivation Base	*F. taipaiensis*	1.8 × 10^4^	1.64	0.72 × 10^6^
Xian Cun, Zhonghoujiao plantation in Lanying Village	*F. taipaiensis*	0.06 × 10^4^	0.07	0.024 × 10^6^
Xian Cun, Balicao plantation in Lanying Village	*F. taipaiensis*	0.08 × 10^4^	0.075	0.032 × 10^6^

### Total alkaloid content determination

**Figure 5:**
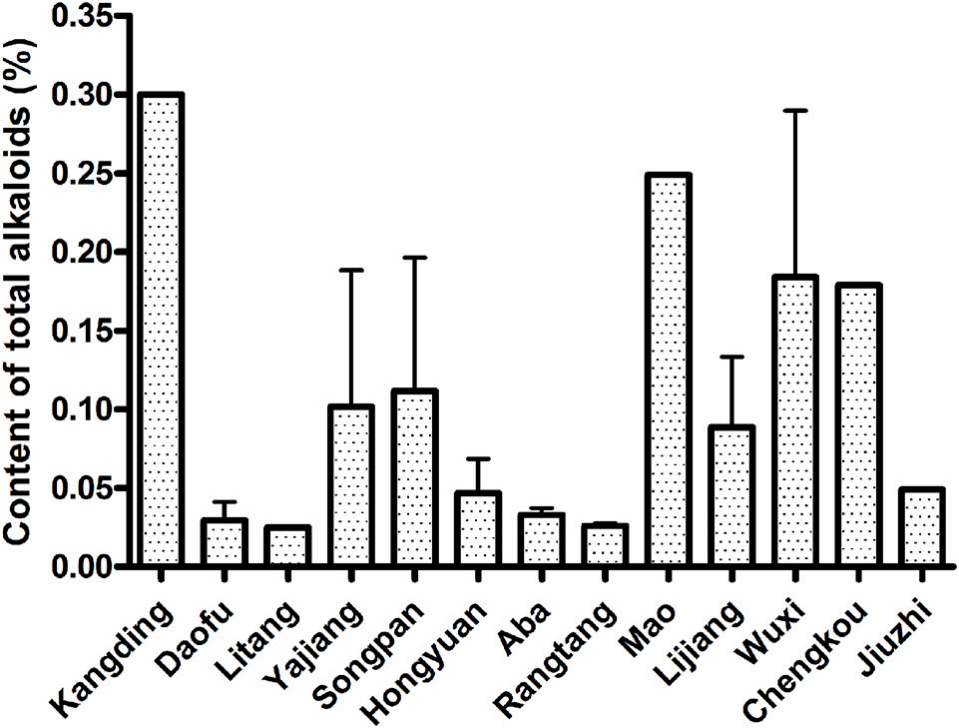
The content of total alkaloids in different traditional producing regions in Qinghai-Tibet Plateau.

The calibration curve was drawn with six standard solutions at concentrations ranging from 0 to 10.43 μg/ml. The calibration curve showed a good linearity with the correlation coefficient being *r* = 0.9984. The regression equation was Y = 17.5668X + 0.1477, where Y and X correspond to the concentration and absorbance, respectively. The data about the content of total alkaloid in different samples are listed in Figure 5 and Table 1. The results show that samples from Mingzhong Village in Chengkou county, Huangcaoping Chuanbeimu Cultivation Base in Wuxi county, Chongqing province and from Enwei Plateau Medicinal Plant Fostering Base in Kangding county, New Lotus Chuanbeimu Cultivation Base in Songpan and Mao counties, Sichuan province have the higher content of total alkaloids. These samples are from artificial cultivation bases or individual plantation, which suggests that the content of total alkaloid of bulbs from artificial cultivation is higher than that from wild fields. The reason is unknown so far.

## Discussion and conclusion

In this investigation, we chose 32 traditional producing regions in Qinghai-Tibet Plateau. There were five traditional producing regions where we did not find the original plants of BFC. In the other investigation sites, the average density of plants is lower than 7, except the site in Ningyou Village in Jiuzhi county, Qinghai province. At the same time, the average resource density is less than 22 mg/m^2^, except for sites in Lasang Mountain in Milong Village in Yajiang county and Ningyou Village in Jiuzhi county. These results indicate that the wild resource of BFC is still at the risk of depletion. The total alkaloid contents in the BFC from Kangding, Mao, Wuxi, and Chengkou are higher than that from other traditional producing regions (Figure 5). In these regions, there are some artificial cultivation bases or individual plantations, where content of total alkaloids of bulbs is higher compared to wild fields. The total alkaloid contents in the BFC from Daofu, Litang, Aba, and Rangtang are much lower than that from other traditional producing regions (Figure 5). The reason is unknown so far.

In order to ensure sustainable utilization of BFC wild resource, the plants of BFC in the 1980s were classified as third level of protection medicinal plants in regulations on the protection of wild medicinal herb resources established by the Chinese government (Liu et al., 2008). Later in 2012, the plants of BFC were classified as precious, rare and threatened species by Endangered Species Scientific Commission of China. Strategies for resource conservation could include regulation, additional cultivation, harvesting after sexual maturity, investigation of micro-propagation, and natural fostering. For the government, establishment of protective regulation is actually one of the most effective strategies for the conservation of wild plants of BFC. At present, the local authorities are trying to perform programs for the propagation and recovering of populations in collaboration with some traditional Chinese medicinal companies and some research groups. Traditional medicinal plants cultivation companies could increase the area of cultivation of the plants of BFC. In addition, cultivation specialist of plant of BFC could conduct training amongst farmers to cultivate the plants of BFC. Medicinal farmers could harvest the BFC after sexual maturity of the plants, which can would help to sustain populations. Researchers could focus on micro-propagation methods like plant tissue and cell culture. Recently, plant tissue culture methods of *F. cirrhosa* (Li, 2012), *F. unibracteata* (Li and Wu, 2011), and *F. przewalskii* (Wang, 2008) was established. In recent years, some researchers proposed natural fostering as a cultivation type of traditional Chinese medicinal plants. They selected *F. cirrhosa* to carry out natural fostering and found natural fostering not only increased local BFC production but also conserved local biological diversity (Li et al., 2012). These approaches could have positive effects to resolve the current contradiction between resource protection and resource utilization. In Europe, there are lots of *Fritillaria* species. Identifying the European closest relatives of the *Fritillaria* species used in traditional medicine could point to additional species that might be analyzed for their potential medicinal value, which may in turn reduce pressure on those species that are currently being collected intensively (Day et al., 2014).

In conclusion, we performed resource investigation in 32 traditional producing regions and found that the wild resource of BFC is at the risk of depletion. In addition, habitat types, plant morphology, and artificial cultivation were investigated and discussed. Lastly, the results of determination of total alkaloid content of samples from different sites display that total alkaloid content of bulbs from cultivation is higher than that of bulbs form wild fields. Artificially cultivated plants of BFC could be an important way to resolve the current contradiction between resource protection and resource utilization. In addition, identifying the European closest relatives of the *Fritillaria* species used in traditional medicine may contribute to resolve this contradiction.

## Author contributions

DW and XC were involved in the initial design of the study, carried out field investigation, and artificial cultivation survey, collected plant specimens and bulbs of BFC and measured the total alkaloid contents of samples. AA, XY, and DW performed data analysis and drafted the manuscript. SW participated in experiments' design and helped to draft the manuscript. All authors read and approved the final manuscript.

### Conflict of interest statement

The authors declare that the research was conducted in the absence of any commercial or financial relationships that could be construed as a potential conflict of interest.

## Abbreviations

BFC: Bulbus Fritillariae Cirrhosae.
